# Comparison of Health Parameters in Postpartum Diastasis Recti: A Randomized Control Trial of SEMG Biofeedback-Assisted Core Strengthening Exercises with Kinesiotaping vs. Non-Assisted Exercises

**DOI:** 10.3390/healthcare12161567

**Published:** 2024-08-07

**Authors:** Ujala Afzal, Quratulain Saeed, Muhammad Nabeel Anwar, Sanna Pervaiz, Manahil Shahid, Rimsha Javed, Muhammad Umair Ali, Seung Won Lee

**Affiliations:** 1 Foundation University College of Physical Therapy, Foundation University Islamabad, Islamabad 44000, Pakistan; ujalai1630@gmail.com (U.A.); quratulain.saeed@fui.edu.pk (Q.S.); spervaiz.phd22smme@student.nust.edu.pk (S.P.); manahil.shahid@fui.edu.pk (M.S.); rjrimsha2@gmail.com (R.J.); 2 School of Mechanical and Manufacturing Engineering (SMME), National University of Sciences and Technology (NUST), Islamabad 44000, Pakistan; nabeel.anwar@smme.nust.edu.pk; 3 Department of Artificial Intelligence and Robotics, Sejong University, Seoul 05006, Republic of Korea; 4 Department of Precision Medicine, Sungkyunkwan University School of Medicine, Suwon 16419, Republic of Korea

**Keywords:** diastasis recti, inter-recti distance, postpartum, biofeedback, surface EMG

## Abstract

Current medical treatments for diastasis recti often involve exercises to strengthen the core muscles, along with abdominal binders or supports. However, there is limited evidence comparing the effectiveness of surface electromyography (SEMG) biofeedback-assisted core strengthening exercises combined with kinesiotaping to other approaches. This study aimed to assess the impact of three interventions on core strength, inter-rectus distance, and quality of life in postpartum women with diastasis recti. The interventions included core strengthening exercises with kinesiotaping and SEMG biofeedback-assisted core strengthening with kinesiotaping. This randomized controlled trial (NCT05897255) included 24 postpartum women divided into three groups. We measured inter-rectus distance, quality of life using the Short Form Health Survey 36, and core strength using the McGill torso battery test. The SEMG biofeedback provided auditory and visual cues. We used one-way analysis of variance to compare outcomes between groups, while a *t*-test for within-group analysis. Both the SEMG biofeedback-assisted and non-assisted core strengthening exercises with kinesiotaping groups showed significantly greater improvements in energy, bodily pain, general health, physical functioning, and limitations due to physical problems than the core strengthening group. Additionally, the SEMG biofeedback-assisted group demonstrated a greater reduction in inter-rectus distance. There were no statistically significant differences in core strength improvement among the three groups. Core strengthening exercises with SEMG-assisted kinesiotaping were superior to core strengthening alone in reducing inter-rectus distance, enhancing physical function, energy levels, and general health, and decreasing bodily pain and limitations due to physical problems. Core strength improvements were similar across all groups.

## 1. Introduction

Diastasis recti abdominis (DRA) is a condition characterized by the separation of the rectus abdominis muscles. The separation occurs at the midline of the linea alba. DRA is a frequent occurrence during pregnancy and the immediate postpartum period. Studies suggest that nearly 70% of women experience it in their third trimester [1]. The separation often persists for up to 6 weeks postpartum, affecting 50–60% of women. Additionally, a significant proportion (approximately 40%) of women may still have DRA at 6 months postpartum, highlighting the potential for a prolonged healing process [2,3]. The majority of these postpartum females suffer from weaker abdominal strength [4,5]. Besides low back pain [6], poor lumbo-pelvic stability, pelvic dysfunction, and urogynecological symptoms [7], if DRA progresses, it can also lead to serious complications requiring medical and surgical interventions. E.g., ventral hernia or divarication of rectus abdominis, infection, etc. [8]. Thus, early diagnosis of DRA and appropriate management are crucial. 

Conservative treatment of DRA in postpartum females remains insufficient in reducing inter-rectus distance (IRD). Although evidence supports its beneficial effects on physical and mental health [9,10]. Certain physical therapy management regimes can cause a limited reduction in IRD, but their impact on patients’ quality of life and functionality needs to be explored [11]. However, exercise targeting various components of core strengthening is effective in reducing IRD. These exercises are patient-customized and safe, i.e., do not report any worsening [4]. A study advocates its execution as early as DRA is diagnosed [12]. Evidence supports that core strengthening can be beneficial in reducing IRD in postpartum DRA without hernia [13]. 

A study by Glupp et al. showed that head lift practice and curl-up exercise improved the thickness and strength of abdominal muscles; however, they did not improve IRD, pelvic floor, or low back disorders in postpartum DRA [14]. Results were justified as the whole core remained untargeted in their study. Core strengthening improves thickness of transverse abdominis and rectus abdominis in primiparous postpartum DRA, which is clinically co-related as activation of transverse abdominis is necessary pre-requisite for implementation of all core strengthening exercises [15]. For optimal performance, strengthening of all core muscle groups, i.e., trunk flexors, extensors, and bilateral side flexors, should be incorporated in the management of DRA, which is lacking in previous literature [13,14,15,16,17]. 

Considering a holistic approach, abdominal binders play a significant role in the management of postpartum DRA [16]. Different other approaches have been used, e.g., core strengthening with corset can provide abdominal support and may improve pain, IRD, and trunk flexion strength [18]. Limited evidence also recommends the use of kinesiotaping (KT) as abdominal bracing, which may improve lumbopelvic stability and function, resulting in decreasing pain [19]. These studies have limitations as only immediate effects of KT have been investigated [19,20]. Moreover, as KT was applied to abdominal muscles, its effects on trunk flexors had only been investigated in the literature [19,21], but core muscles work in coordination [22], and thus the effects of KT on other core muscles also need to be explored. This study has investigated the long-term effects of KT considering all core muscle groups, thus filling the research gap.

Lastly, while SEMG biofeedback was traditionally used in pelvic floor therapy for overactive bladders [23], recent studies have explored its use for targeting pelvic floor muscles in postpartum women with DRA [24]. A study used intravaginal electrodes for biofeedback-assisted pelvic floor muscle training, which was effective in reducing IRD and improving quality of life in DRA [24]. A more robust methodology could have incorporated biofeedback from the abdominal muscles, e.g., rectus abdominis, as its weakness and separation from linea alba in the primary cause of DRA. A study showed that neuromuscular electrical stimulation (NMES) of the rectus abdominis with core strengthening was better than kinesiotaping with core strengthening in the management of DRA in reducing IRD and improving trunk flexor strength [25]. This raises the question of whether SEMG biofeedback-assisted core strengthening without NMES could be effective in managing DRA. As the central dogma of core strengthening is the activation of transversus abdominis, incorporated strengthening of all core muscle groups [22] and additional auditory and visual stimuli through SEMG biofeedback from abdominal muscles can possibly facilitate the patient’s efficient activation. However, none of the previous literature has investigated this effect. In this study, we have addressed it by investigating the effects of SEMG biofeedback-assisted core strengthening in postpartum DRA.

Although the literature emphasizes and provides evidence of beneficial effects of core strengthening along with usage of abdominal support, e.g., kinesiotaping [8,10,12], there is limited literature using SEMG biofeedback from abdominal muscles for core strengthening in postpartum females with DRA.

To our knowledge, no prior research has compared core strengthening treatment with these alternative techniques: core strengthening with KT and core strengthening with KT along with SEMG biofeedback without electrical stimulation. This study addresses the question of whether there will be a difference between SEMG biofeedback-assisted versus non-assisted core strengthening with kinesiotaping on health parameters such as core strength, inter-rectus distance, and quality of life in postpartum females with diastasis recti. 

## 2. Materials and Methods

### 2.1. Study Design

A parallel-design randomized controlled trial was conducted to investigate the effectiveness of different interventions for DRA in postpartum women. Participants were recruited from the community of Rawalpindi District, Pakistan, through online social media invitations and referrals from the gynecological, obstetric, and rehabilitation departments of Fauji Foundation Hospital from June 2022 to July 2023. 

### 2.2. Ethics

The study received ethical approval (FF/FUMC/215-274Phy/23) from the ethical review committee of the Foundation University School of Health Sciences. The research was conducted in accordance with the Declaration of Helsinki and CONSORT guidelines. Prospective registration with clinicaltrials.gov (National Institute of Health, US) ensured transparency (ID: NCT05897255) as recommended by CONSORT and ICMJE. 

Participants were thoroughly briefed on the study details, including the confidentiality of their information. They received a clear explanation of SEMG biofeedback, core strengthening exercises, KT, and how these interventions aim to improve inter-rectus distance (IRD), abdominal strength, and quality of life. All participants provided written informed consent following the Declaration of Helsinki to participate in the study. Additionally, a small number of participants consented to have their images taken during treatment.

### 2.3. Sample Size 

A sample size of 24 participants (eight women per group) was determined using G* Power software version 3.1. This calculation considered a large effect size (0.7), an alpha level of 0.05, and a power set of 0.80. This sample allowed us to statistically detect a group difference in core muscle strength between women exercising with KT, with and without SEMG biofeedback. 

### 2.4. Participant Selection and Random Allocation

Thirty-five postpartum women with DRA were initially assessed for eligibility. Following a non-probability purposive sampling approach, 24 participants met the inclusion criteria and were randomly assigned to one of three groups. The inclusion criteria were as follows: women aged 18–40 years, 6–24 months postpartum, diastasis recti distance >2 cm at any of the three levels measured, and primiparous or multiparous with spontaneous vaginal delivery or lower section cesarean section. Exclusion criteria were as follows: skin sensitivity to taping materials; open abdominal wounds or skin diseases; presence of abdominal hernia; prior abdominal or back surgical; heart or respiratory conditions; neurological diseases; musculoskeletal disorders in the upper and lower extremities; current pregnancy; and lower back pain intensity exceeding a value of 8 on the numeric pain rating scale. 

**Figure 1:**
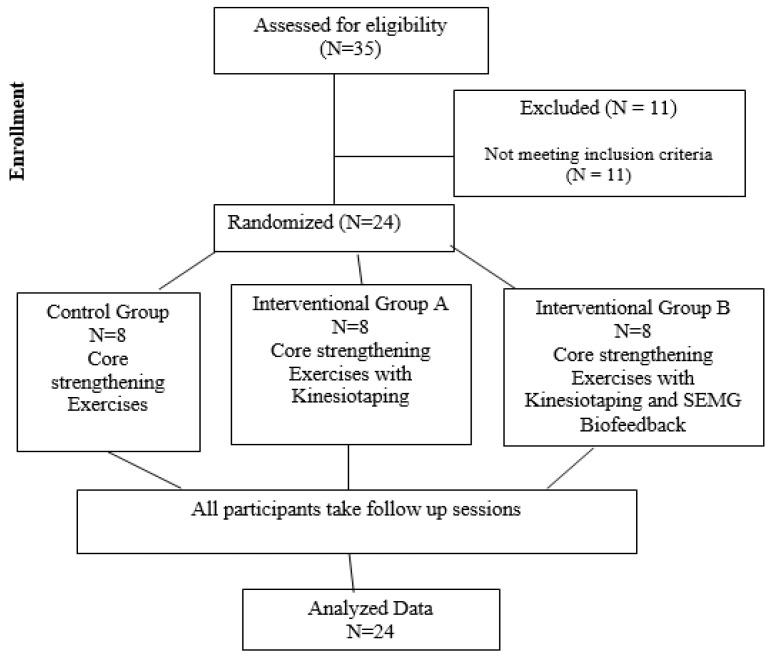
Scheme of the study (consort diagram).

Following enrollment, participants were randomly allocated to one of three groups using the sealed envelope method (i.e., participants were asked to choose one of the sealed envelopes and were allocated accordingly): the core strengthening exercise group (control group), core strengthening exercises with the KT (Interventional A) group, and SEMG biofeedback-assisted core strengthening exercises with the KT (Interventional B) group (Figure 1). This study was single-blinded (i.e., participants were unaware of the allocated group).

### 2.5. Intervention

All groups participated in a core strengthening exercise program that included abdominal tuck-in, supine marching, supine straight leg raises (SLRs), supine bridges, partial curl-ups, standing wall squats, planks, and quadruped reciprocal upper extremity and lower extremity raises (Birddog exercise). Participants were given rest periods between exercises. Initially, the target given to participants was to perform two sets of five repetitions of each exercise, including abdominal hollowing, curl-ups, sit-ups, pelvic bridging, and SLRs. Abdominal hollowing was a necessary component of all exercise, as activation of transverse abdominis is the central idea during the performance of all exercises. Once this target was achieved, gradually exercises were added; repetitions and hold time were increased according to the participant as described by the standardized protocol of Hick et al. (i.e., intensity can be increased maximum up to 8 s hold; 30 repetitions) [26]. The number of repetitions and hold times for each exercise varied depending on the exercise intensity each participant can perform. This variation was managed as all participants were given the time duration of 30–45 min to complete their session.

The control group was allowed to use abdominal binder/brace when they felt the need for support during functional activities, but it was not worn during exercise sessions. Whereas intervention group A performed the core strengthening exercise as described above with the addition of KT taping. Intervention group B performed the core strengthening exercises with KT taping and received additional real-time feedback on their muscle activity through SEMG biofeedback. KT remained applied for up to 24 h on participants of groups A and B; thus, they performed their functional activities with KT applied. None of the participants reported any adverse effects of KT. All participants received the assigned intervention three times a week for 6 weeks.

All participants of intervention groups A and B received KT with two Y-shaped tapes, regardless of their assigned group. KT was applied while the patient was in a standing position. Each tape was approximately 20 cm long. The base of the first tape was placed on the middle section of the external oblique without tension. One tail of the Y-tape was applied over the navel with 20% tension. The other tail was applied below the navel with 20% tension. These steps were repeated on the opposite side of the abdomen using a second piece of tape. 

### 2.6. SEMG Feedback

**Figure 2:**
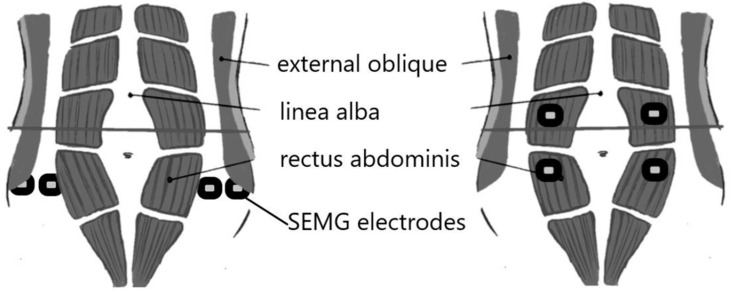
SEMG electrode placements on rectus abdominis and external oblique.

Participants in Interventional group B received core strengthening exercises along with real-time feedback on their muscle activity through SEMG biofeedback (Figure 2). During exercise, SEMG electrodes recorded electrical activity through bipolar electrodes from the rectus abdominis and external oblique muscles (Figure 2).

The SEMG Biofeedback system was calibrated before each session for every recruited patient of the group. Participants performed a series of 10-second muscle contractions with ten seconds of rest in between each contraction for a minute. This activity was performed prior to each session to record maximum voluntary contraction (MVC), which was taken as a reference for that particular training session. In order to prevent muscle fatigue during session, the target contraction intensity ranged from 10 to 50% of the participant’s maximal voluntary contraction. If needed, participants could start with shorter work/rest intervals (e.g., 3-second contraction and 6-second rest) and gradually increase the duration as they build endurance. 

**Figure 3:**
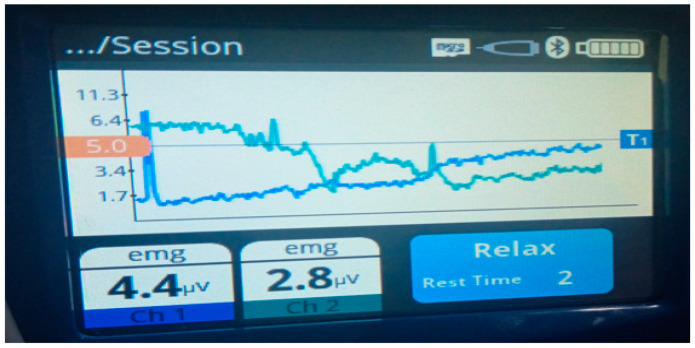
SEMG biofeedback device indicating muscle relaxation below 5 μV.

During treatment, the screen displayed a representation of the measured and amplified electrical activity from the muscles in graphical form, depicting the target value as a horizontal line. The screen displayed the icon “contract”, after which the patient contracted the muscle, which resulted in increasing the amplitude of the graph. Patients were required to raise and maintain the peak above threshold value for specified seconds customized according to patient (e.g., 3, 6, and 10). As the patient contracted, a tone sounds, giving a positive biofeedback to the patient. The frequency of sound beeps increased with intensity of muscle contraction, which was also being displayed on screen in mV. Next, the icon of “relax” appears, and the patient was trained to decrease the peak below 5 μV (Figure 3). 

This process was repeated for a determined number of repetitions (trials) in a session, starting with a smaller number of repetitions, e.g., 5, then gradually increasing. After successful completion of repetitions, a thumbs-up sign appears on the screen. The same standard protocol of initial two sets of five repetitions was performed by the participants, which was gradually increased as per participants’ tolerance. We utilized a maximum of two channels at a time. Signals from each channel were displayed in different colors on screen. This facilitated simultaneous training of muscles bilaterally. Parameters of targeted muscle contraction, i.e., intensity/peak voltage, contraction time duration, relaxation time, and number of repetitions, were customized according to the patient. This combined audio–visual feedback aimed to encourage and motivate participants to maintain proper activation during exercise.

### 2.7. Outcome Measures

As per literature, we found discrepancy in response of inter-rectus distance and core strength assessment to different exercise regimes of core strengthening and bracing, i.e., IRD decreased in some studies while remained the same in others. Similarly, the core strength of some muscle groups improved while others remained unchanged [14,18,27].

Since the exercise protocol of this study incorporated all essential core muscle strengthening, we hypothesize that it must decrease IRD at all levels. Therefore, a mechanical dial Vernier caliper was used to measure the IRD at three levels: the umbilicus level, 2 cm above the umbilicus, and 2 cm below the umbilicus. Participants were positioned lying on their back with a slight bend at the knees (hook-lying position). The caliper jaws were opened and placed on the outer edges of the recti abdominis muscles at each measurement point. To activate the abdominal muscles and ensure accurate measurements, participants performed a partial curl-up. The Vernier calipers are known for their high reliability, with intra-class correlation coefficients (ICC) ranging from 0.80 to 0.99 and a retest reliability ICC of 1.00 [28]. 

The Short-Form 36 Health Survey (SF-36) is a validated tool used to assess a person’s health perception across eight domains: physical functioning, physical role functioning, bodily pain, general health perceptions, mental health, social functioning, and energy levels. Higher scores on the SF-36 indicate better health. The SF-36 used in this study demonstrated strong internal consistency with a Spearman–Brown coefficient of 0.828 and a Cronbach’s alpha of 0.838.

Since the strengthening protocol of previous literature was either focused on any one core muscular group, e.g., trunk flexors [14,27], or was focused on only one sub-group of postpartum diastasis patients, e.g., primiparous [15], there was variation in the literature of core strength improvement. In this study, all participants performed core strengthening of all muscles. McGill’s torso muscular endurance test battery was used to evaluate core strength. This test battery consists of four components: trunk flexion endurance test, trunk extension endurance test, and right and left lateral endurance tests. Each test was repeated three times with two minutes of rest between repetitions. The average time for each test was recorded. McGill’s torso muscular endurance test is known to have good reliability, with an ICC > 0.82. 

Measurements were taken at two time points: pre-treatment (before the beginning of sessions) and post-treatment (i.e., after completing 18 treatment sessions). 

### 2.8. Statistical Analysis

Data analysis was performed using the Statistical Package for Social Sciences v21.0. A 95% confidence interval was applied, and a *p*-value less than 0.05 was considered statistically significant. The Shapiro–Wilk test showed that data were normally distributed. Descriptive quantitative variables were summarized using means and standard deviations. Qualitative variables were described using frequencies and percentages. A one-way ANOVA was used to compare differences among the three groups at post-treatment on the outcome measures. Tukey’s post hoc was applied to the variables whose *p* value was less than 0.05 for further analysis in order to determine which groups exactly differ from each other. A paired *t*-test was applied to determine the statistical difference between pre-treatment and post-treatment values within each group.

## 3. Results

A total of 35 women were initially assessed for eligibility, and 24 (68.5%) met the inclusion criteria and were enrolled in the study. All participants completed the treatment program (Figure 1). The average age of participants was 27.4 ± 4.93 years. There were no significant age differences between the groups (Table 1). Most participants (70.8%, *n* = 17) had vaginal deliveries, while the remaining participants (29.2%, *n* = 7) had cesarean sections. In terms of post-treatment comparison, a significant difference was observed between the three treatment groups in terms of quality of life (physical functioning, limitations due to physical problems, energy, body pain, and general health, *p* < 0.05) and IRD at and above the umbilicus level. No significant differences in core strength, inter-rectus distance below the umbilicus level, or the remaining sub-division of quality of life (*p* > 0.05) were observed between the groups. Overall, significant improvements (*p* < 0.05) were observed in several quality of life sub-scores and IRD measurements after treatment compared to pre-treatment values. However, the effect differed between the intervention groups (Table 1). 

There were no significant differences in quality of life between groups A and B (*p* > 0.05). Both groups showed improvements in physical functioning, limitations due to physical problems, energy, body pain, and general health compared to the control group (Table 2). IRD at and above the umbilicus level significantly decreased in the control group and intervention group B after treatment but not in intervention group A (Table 2).

When looking at each group individually (pre- and post-treatment), all groups showed statistically significant improvements (*p* < 0.05) on all outcome measures, except for social functioning, role limitations due to emotional problems, and role limitations due to physical problems (*p* > 0.05) (Table 3).

## 4. Discussion

This study investigated the effectiveness of different interventions for DRA in postpartum women. The interventions compared were as follows: core strengthening exercises, core strengthening exercises with KT, and SEMG biofeedback-assisted core strengthening exercises with KT. This study examined the effects of these interventions on IRD, core strength, and quality of life. 

All three groups showed a general improvement in IRD, which aligns with previous research suggesting that abdominal bracing with core strengthening is effective in reducing DRA [18]. Results are aligned with the meta-analysis, which concluded that KT is effective in improving IRD above umbilicus [29]. In our study, the improvements were significantly better at and above the umbilicus in intervention group B. This suggests that incorporating SEMG biofeedback with core exercises and KT may offer superior benefits in reducing DRA. Possible reasons could be as follows: (a) KT remained applied even during performance of core strengthening, which provides stability and proprioceptive stimuli [30]; (b) reinforces trunk flexors to work in coordination [30] as it was applied as two Y-shaped strips; and (c) contributes to correcting the biomechanics [30] not only during exercise sessions but also during functional activities as it remained applied for 24 h. Thus, group B received additional three stimuli during core strengthening, i.e., proprioceptive, auditory, and visual. Simultaneously, facts from the results also showed that although core strengthening improved IRD below umbilicus level in all three groups, there was no difference among them. Which implicates that SEMG-assisted core strengthening with KT can be a better choice of treatment in patients presenting with above and at umbilical DRA, while if the patients present with DRA below umbilical level, i.e., above and at umbilical level, have been managed, the solely core strengthening protocol would be sufficient to gain similar results. 

Patient with DRA presents with poor lumbopelvic stability [6], which indicates the strength of the abdominal musculature is influenced by IRD. In this research, all participants not only showed improved IRD at all three levels but also showed improved core strength in all muscle groups, i.e., flexors, extensors, and side flexors. We failed to detect any significant difference among groups. We measured the strength in terms of holding time (seconds) by the positions described by the McGill Torso battery. A recent study demonstrated that NMES combined with core stabilization exercises improved core strength and patient recovery from DRA with fewer side effects like low back pain [25]. Their study assessed muscle strength by manual muscle testing, which requires only one attempt at performance. As the variable of measuring strength in their study is different, we cannot compare and contrast core strength results with their study. Another study used EMG-assisted biofeedback from pelvis floor muscles combined with NMES to rectus abdominis [25], which decreases IRD. Although its theoretical frame was based on principles of biofeedback [31] and improving core muscle strength using electrical stimulation [32], the study lacks the objective of core strength assessment. While our study did not use NMES, the observed improvements in core strength across all groups suggest that SEMG-assisted electrical stimulation of core muscles for DRA warrants further exploration. 

The between-group analysis of quality of life revealed significant improvements in specific sub-domains: physical functioning, energy levels, body pain, and general health. Both intervention groups showed greater improvements in these areas compared to the control group. These results are consistent with previous studies. Biofeedback from the pelvic floor combined with NMES to rectus abdominis muscles showed improvement in the physical domain [24]. Another study showed that core strengthening exercises improve the quality of life in terms of physical functioning in women with DRA [33]. The KT helps bring the separated rectus abdominis muscles closer together, potentially leading to reduced pain and increased energy levels [20]. This might be the underlying phenomenon for the improvement in energy and body pain in both interventional groups compared to that in the control group. However, there were no statistically significant differences between groups nor within groups for role limitations due to emotional problems and social functioning. This might be due to the fact that our intervention protocol lacks a bio-psychosocial component [34], which must be addressed in future studies. This study is the first to examine the impact of SEMG biofeedback on all domains of quality of life in postpartum women with DRA. For effective treatment, a thorough assessment of DRA patients is important so that the sub-domains of quality of life that need to be addressed must be focused holistically to gain an optimal outcome from rehabilitation. 

This current study has a few limitations that are important to consider. The study only involved a relatively small sample size, with only 24 participants divided among three groups. This can limit the statistical power to detect significant differences between groups. Future studies with larger and more diverse participant pools would be beneficial. Second, DRA measurements were obtained manually using a digital caliper. While this method is reliable, more advanced techniques like ultrasound or magnetic resonance imaging scans could potentially provide more precise measurements. It is important, however, that the chosen method does not necessarily invalidate the study’s results. Third, the SEMG biofeedback component of the treatment program was implemented for a short period. Further research is needed to explore the long-term effects of combining electrical stimulation with SEMG biofeedback training, KT, and core-strengthening exercises on DRA. Fourthly, although core strength was measured objectively in the study, our study lacks electromyographic evidence, which could possibly highlight the unexpected results at the electrophysiological level. Certain confounding factors may also influence our results, e.g., (a) participants of the control group were allowed to use braces for support when needed. We were not able to document the type of brace, worn duration, and frequency; (b) quality of life was subjectively assessed, which might have been influenced by the bio-psychosocial component [34]; and (c) although a standardized core strengthening protocol was given, whose initial target was two sets of five repetitions of each exercise; once patients achieved it, they were progressed according to progression criteria but with an individualized pace. We were not able to document if the weekly progression among the three groups was the same. Confounding factors can be managed considering mixed-method research design, weekly diary, and repeated measure of outcome variables. 

## 5. Conclusions

SEMG-assisted core strengthening with KT in postpartum DRA rehabilitation should be treatment goal-oriented, as it can be more beneficial in terms of inter-rectus distance reduction and improving physical functioning, energy levels, and overall general health while improving core strength, similar to the core strengthening protocol.

## Figures and Tables

**Table 1:** Post-treatment inter-group comparison between the control group (core strengthening exercises), Interventional A (core strengthening exercises with kinesiotaping) group, and Interventional B (core strengthening exercises with kinesiotaping + surface electromyography) group in terms of inter-rectus distance, quality of life, and core strength.

Variable	Control Group (Mean ± SD)	C.I 95%	Interventional A (Mean ± SD)	C.I 95%	Interventional B (Mean ± SD)	C.I 95%	F Value	*p*-Value
Age (years)	26.7 ± 5.23	22.37 to 31.12	26.7 ± 4.43	23.04 to 30.45	28.7 ± 5.47	24.18 to 33.32	0.416	0.597
Height (cm)	151.7 ± 3.64	148.67 to 154.77	162.8 ± 5.04	158.65 to 167.08	159.1 ± 8.52	152.04 to 166.31	6.94	0.007 *
Weight (kg)	53.3 ± 11.0	44.14 to 62.61	63.5 ± 8.89	56.06 to 70.94	60.3 ± 12.5	49.87 to 70.88	1.79	0.137
BMI (kg/m^2^)	22.4 ± 2.81	20.07 to 24.78	23.8 ± 2.76	21.49 to 26.11	25.4 ± 4.82	21.42 to 29.48	1.41	0.437
**Inter-rectus distance**								
At umbilicus level (cm)	2.65 ± 0.28	2.42 to 2.89	2.50 ± 0.29	2.26 to 2.74	2.22 ± 0.37	1.91 to 2.54	3.82	0.038 *
Above umbilicus level (cm)	2.74 ± 0.25	2.52 to 2.96	2.56 ± 0.327	2.29 to 2.84	2.28 ± 0.37	1.97 to 2.60	3.96	0.035 *
Below umbilicus level (cm)	2.37 ± 0.19	2.21 to 2.54	2.28 ± 0.29	2.03 to 2.53	2.11 ± 0.39	1.78 to 2.45	1.42	0.262
**Quality of life**								
Role limitation due to physical problems	44.37 ± 25.41	23.13 to 65.62	92.50 ± 11.34	83.02 to 101.97	91.87 ± 15.56	78.86 to 104.89	9.313	0.001 *
Role limitation due to emotional problems	82.27 ± 27.62	59.18 to 105.37	88.75 ± 31.81	62.15 to 115.35	85.83 ± 29.04	61.56 to 110.12	0.096	0.909
Energy	46.25 ± 10.93	37.11 to 55.39	70.62 ± 19.16	54.60 to 86.65	66.25 ± 14.58	54.06 to 78.44	5.79	0.010 *
Physical functioning	65.62 ± 15.45	52.70 to 78.54	91.87 ± 10.99	82.68 to 101.07	90.00 ± 13.88	78.39 to 101.61	9.313	0.001 *
Social functioning	56.12 ± 14.88	44.68 to 69.57	66.81 ± 27.35	44.99 to 90.64	68.12 ± 22.22	56.85 to 92.28	0.710	0.503
Body pain	60.00 ± 20.08	43.21 to 76.79	83.75 ± 12.67	73.15 to 94.35	88.12 ± 13.00	77.25 to 98.99	7.494	0.003 *
General health	57.50 ± 11.95	47.51 to 67.49	81.25 ± 12.74	70.59 to 91.91	80.12 ± 14.74	67.80 to 92.45	8.245	0.002 *
**Core strength**								
Trunk flexion (s)	10.00 ± 2.01	8.31 to 11.68	16.06 ± 7.48	9.86 to 22.26	16.1 ± 6.99	10.25 to 21.95	2.739	0.088
Trunk extension (s)	8.81 ± 2.64	6.60 to 11.02	12.87 ± 4.96	8.73 to 17.02	13.50 ± 6.60	7.98 to 19.02	2.066	0.152
Right side plank (s)	8.37 ± 2.91	5.95 to 10.81	12.10 ± 6.16	6.95 to 17.25	10.18 ± 4.78	6.19 to 14.18	1.201	0.321
Left side plank (s)	8.46 ± 2.7	6.18 to 10.74	11.81 ± 5.7	6.99 to 16.62	10.06 ± 4.78	6.06 to 14.06	1.061	0.364

* *p*-value significant at <0.05; BMI: body mass index; SD: standard deviation; C.I. 95%: confidence interval at 95%.

**Table 2:** Post hoc analysis using Tukey’s test.

Variable	Groups	Mean ± SD	Mean Δ	C.I 95%	*p*-Value
At umbilicus level	Control	2.65 ± 0.28	0.154	−2.472 to 0.555	0.605
	Intervention A	2.50 ± 0.29			
	Control	2.65 ± 0.28	0.433	0.032 to 0.835	0.032 *
	Intervention B	2.22 ± 0.37			
	Intervention A	2.50 ± 0.29	0.280	−1.209 to 0.680	0.207
	Intervention B	2.22 ± 0.37			
Above umbilicus level	Control	2.74 ± 0.25	0.175	−2.335 to 0.583	0.537
	Intervention A	2.56 ± 0.327			
	Control	2.74 ± 0.25	0.452	0.044 to 0.861	0.028 *
	Intervention B	2.28 ± 0.37			
	Intervention A	2.56 ± 0.327	0.277	−0.131 to 0.686	0.224
	Intervention B	2.28 ± 0.37			
Physical functioning	Control	65.62 ± 15.45	−26.250	−43.356 to −9.144	0.002 *
	Intervention A	91.87 ± 10.99			
	Control	65.62 ± 15.45	−24.375	−41.480 to −7.269	0.005 *
	Intervention B	90.00 ± 13.88			
	Intervention A	91.87 ± 10.99	1.87	−15.230 to 18.980	0.959
	Intervention B	90.00 ± 13.88			
Role limitations due to physical problems	Control	44.37 ± 25.41	−48.125	−71.329 to −22.921	0.012 *
	Intervention A	92.50 ± 11.34			
	Control	44.37 ± 25.41	−47.500	−70.703 to −24.296	0.006 *
	Intervention B	91.87 ± 15.56			
	Intervention A	92.50 ± 11.34	0.625	−22.57 to 23.829	0.952
	Intervention B	91.87 ± 15.56			
Energy	Control	46.25 ± 10.93	−24.375	−43.620 to −5.131	0.012 *
	Intervention A	70.62 ± 19.16			
	Control	46.25 ± 10.93	−20.000	−39.245 to −0.755	0.041 *
	Intervention B	66.25 ± 14.58			
	Intervention A	70.62 ± 19.16	4.375	−14.870 to 23.620	0.836
	Intervention B	66.25 ± 14.58			
Body pain	Control	60.00 ± 20.08	−23.750	−43.456 to −4.044	0.017 *
	Intervention A	83.75 ± 12.67			
	Control	60.00 ± 20.08	−28.125	−47.831 to −8.419	0.005 *
	Intervention B	88.12 ± 13.00			
	Intervention A	83.75 ± 12.67	−4.375	−24.081 to 15.331	0.843
	Intervention B	88.12 ± 13.00			
General health	Control	57.50 ± 11.95	−23.750	−40.384 to −7.116	0.005 *
	Intervention A	81.25 ± 12.74			
	Control	57.50 ± 11.95	−22.625	−39.259 to −5.990	0.007 *
	Intervention B	80.12 ± 14.74			
	Intervention A	81.25 ± 12.74	1.125	−15.509 to 17.759	0.984
	Intervention B	80.12 ± 14.74			

* *p*-value significant at <0.05; SD, standard deviation.

**Table 3:** Intra-group comparison in terms of inter-rectus distance, quality of life, and core strength.

Variables		Time	Control Group				Interventional Group A				Interventional Group B			
			Mean ± SD	Mean Δ	C.I 95%	*p*-Value	Mean ± SD	Mean Δ	C.I 95%	*p*-Value	Mean ± SD	Mean Δ	C.I 95%	*p*-Value
Inter-rectus distance	At umbilicus level	Pre Rx	2.74 ± 0.31	0.086	0.027 to 0.144	0.010	2.99 ± 0.300	0.491	0.370 to 0.611	0.000	2.85 ± 0.40	0.633	0.365 to 0.902	0.001 *
		Post Rx	2.6 ± 0.28				2.50 ± 0.29				2.22 ± 0.37			
	Above umbilicus level	Pre Rx	2.95 ± 0.28	0.215	0.131 to 0.298	0.001	3.20 ± 0.28	0.642	0.246 to 0.533	0.000	3.07 ± 0.34	0.781	0.546 to 1.015	0.000 *
		Post Rx	2.74 ± 0.25				2.56 ± 0.33				2.28 ± 0.37			
	Below umbilicus level	Pre Rx	2.45 ± 0.25	0.0762	0.024 to 0.127	0.010	2.67 ± 0.38	0.390	0.246 to 9.533	0.000	2.59 ± 0.40	0.477	0.199 to 0.755	0.005 *
		Post Rx	2.37 ± 1.98				2.28 ± 0.29				2.11 ± 0.39			
Core Strength	Trunk Flexion	Pre Rx	7.47 ± 2.27	−2.52	−3.325 to −1.172	0.000	11.61 ± 7.39	−4.450	−5.592 to −3.307	0.000	10.04 ± 6.35	−6.062	−7.429 to −4.696	0.000 *
		Post Rx	10.0 ± 2.01				16.06 ± 7.41				16.10 ± 6.99			
	Trunk Extension	Pre Rx	5.97 ± 2.52	−2.83	−3.842 to −1.832	0.000	7.65 ± 4.57	−5.225	−5.793 to −4.656	0.000	6.79 ± 5.93	−6.71	−9.109 to −4.315	0.000 *
		Post Rx	8.81 ± 2.64				12.87 ± 4.96				13.50 ± 6.60			
	Right side planks	Pre Rx	6.18 ± 2.86	−2.18	−3.002 to −1.372	0.000	7.56 ± 5.45	−4.537	−5.755 to −3.319	0.000	4.04 ± 3.74	−6.150	−7.443 to −4.855	0.000 *
		Post Rx	8.37 ± 2.91				12.1 ± 6.16				10.19 ± 4.78			
	Left side planks	Pre Rx	6.31 ± 2.69	−2.15	−2.635 to −1.664	0.000	7.85 ± 5.64	−3.96	−4.968 to −2.956	0.000	3.37 ± 3.25	−6.687	−8.864 to −4.510	0.000 *
		Post Rx	8.46 ± 2.73				11.81 ± 5.76				10.06 ± 4.78			
Quality of life	Physical functioning	Pre Rx	59.37 ± 18.01	−6.250	−9.955 to −2.544	0.005	82.50 ± 16.69	−9.375	−14.585 to −4.164	0.004	77.0 ± 23.71	−13.000	−22.288 to −3.711	0.013 *
		Post Rx	69.62 ± 15.45				91.87 ± 10.99				18.0 ± 13.89			
	Social functioning	Pre Rx	56.12 ± 14.88	−1.000	−2.611 to 0.611	0.186	66.81 ± 27.35	−1.000	−2.611 to 0.6112	0.186	68.12 ± 22.22	−6.437	−17.184 to 4.309	0.200
		Post Rx	57.12 ± 14.88				67.81 ± 27.30				74.56 ± 21.18			
	Role limitations due to emotional problems	Pre Rx	75.0 ± 38.83	−7.275	−18.684 to 4.13	0.175	83.34 ± 35.63	−5.412	−15.276 to 4.451	0.236	79.16 ± 36.20	−6.675	−16.460 to 3.11	0.151
		Post Rx	82.27 ± 27.63				88.75 ± 31.81				85.83 ± 29.04			
	Role limitations due to physical problems	Pre Rx	15.65 ± 35.18	−28.718	−42.038 to −15.398	0.001	75.62 ± 40.12	−16.875	−41.242 to 7.492	0.146	81.25 ± 37.20	−10.000	−32.56 to 11.316	0.290
		Post Rx	44.37 ± 25.41				92.50 ± 11.34				91.87 ± 15.57			
	Energy	Pre Rx	43.12 ± 9.23	−9.312	−24.71 to 6.091	0.011	60.62 ± 24.70	−10.000	−16.31 to −3.68	0.007	47.50 ± 15.58	−18.750	−22.455 to −15.044	0.000 *
		Post Rx	46.25 ± 10.94				70.62 ± 19.17				66.25 ± 14.57			
	Body pain	Pre Rx	53.06 ± 20.03	−6.937	−10.543 to −3.331	0.003	72.94 ± 16.98	−10.81	−16.221 to −5.403	0.002	77.37 ± 22.67	−10.750	−20.391 to −1.108	0.034 *
		Post Rx	60.0 ± 20.09				83.73 ± 12.67				88.12 ± 13.0			
	General health	Pre Rx	50.0 ± 15.12	−7.500	−10.659 to −4.340	0.001	71.25 ± 14.07	−10.000	−13.870 to −6.129	0.000	64.12 ± 16.61	−16.000	−21.399 to −10.600	0.000 *
		Post Rx	57.5 ± 11.95				61.25 ± 12.75				80.12 ± 14.74			

* *p*-value significant at <0.05; Pre Rx: pre-treatment; Post Rx: post-treatment; Mean Δ: mean difference.

## Data Availability

The data were confined to the researcher, and the confidentiality of the participants was maintained. The raw data supporting the conclusions of this article will be made available by the authors upon reasonable request via email.
